# Ellis–van Creveld Syndrome in Iran, a Case Report and Review of Disease Cases in Iran, Middle East

**DOI:** 10.15388/Amed.2021.28.2.11

**Published:** 2021-08-20

**Authors:** Behnam Baghianimoghadam, Aidin Arabzadeh, Yousef Fallah

**Affiliations:** Imam Khomeini Hospital Complex, Department of Orthopedic Surgery, Tehran University of Medical Sciences, Tehran, Iran; Imam Khomeini Hospital Complex, Department of Orthopedic Surgery, Tehran University of Medical Sciences, Tehran, Iran; Sina Hospital, Department of Orthopedic Surgery, Tehran University of Medical Sciences, Tehran, Iran

**Keywords:** Ellis-van Creveld, Iran

## Abstract

**Introduction:**

Chondroectodermal dysplasia (Ellis–van Creveld syndrome (EVC)) (OMIM: **#**225500) is a rare skeletal dysplasia with unknown exact prevalence. EVC patients may have abnormal skeletal and extra skeletal symptoms. We report a case of EVC patient and review previous cases reported in Iran.

**Materials and methods:**

The patient was a 30 years old woman referred to our hand clinic for an extra finger in both hands. She was born to a consanguineous marriage. The patient had a history of bilateral valgus deformity of the knees, for which she underwent proximal tibial and distal femoral osteotomy. Upon examination, the patient had bilateral polydactyly and brachymetacarpia with hypotrophic fingernails. She was about 120 cm tall and had acromesomelic type dwarfism. Oral examination revealed serrated incisal margins, dental transposition, a diastema, conical teeth and, enamel hypoplasia. In the radiographic examination of upper extremity, postaxial polydactyly and polymetacarpia, enlarged distal radius, and fusion of capitate and hamate were seen. We searched online databases (Pubmed, Scopus, Google Scholar) and found 14 Iranian papers with 21 reported patients (there was no time limit). We reviewed available clinical and genetic data and the geographic origins of patients.

**Results:**

14 articles reporting 22 EVC patients (including our patient) from Iran have been published in Persian and English (7 in Persian and 7 in English). All patients presented with characteristic EVC symptoms but were diagnosed at a relatively late age, 18 patients were born to consanguineous marriages

**Conclusions:**

It seems that based on these studies, it is possible to identify some families with this genetic mutation. We can warn such families of the dangers of consanguineous marriage through genetic counseling before marriage. In addition, by identifying families with such problems, we can detect such anomalies in the baby earlier with more careful prenatal care.

## Abstract (lt)

**Ellis-van Creveld sindromas Irane – pranešimas apie atvejį ir ligos atvejų apžvalga Irane, vidurio rytuose:**

**Santrauka. *****Įžanga.*** Chondroektoderminė displazija (Ellis-van Creveld sindromas (EVC)) (įrašas Mendelio žmonių paveldimumo tinklalapyje (OMIM): #225500) – reta skeleto displazija, tikslaus jos paplitimo masto nėra nustatyta. Pacientams, kuriems nustatytas EVC sindromas, gali pasireikšti nenormalūs skeleto ir minkštųjų audinių simptomai. Aprašome pacientės, kuriai nustatytas EVC, atvejį ir apžvelgiame ankstesnius atvejus, nustatytus Irane.

***Medžiaga ir metodai.*** Pacientė – 30 metų moteris, kuri gavo siuntimą į mūsų rankų chirurgijos kliniką dėl papildomo piršto kiekvienoje plaštakoje. Ji gimė santuokoje, sudarytoje tarp artimų giminaičių. Pacientei anksčiau nustatyta abiejų kelių *valgus* tipo deformacija, todėl jai atlikta proksimalinio blauzdikaulio ir distalinio šlaunikaulio osteotomija. Apžiūros metu pacientei nustatyta abiejų plaštakų daugiapirštystė ir hipotrofiniai pirštų nagai. Pacientės ūgis – apie 120 cm, jai nustatytas hipofizinis nanizmas. Burnos apžiūros metu nustatyta dantytas priekinių dantų kraštas, dantų transpozicija, kūgio formos dantys ir emalio hipoplazija. Padarius rankų rentgeno nuotrauką, nustatyta ulinarinė polidaktilija, polimetakarpija, padidėjęs distalinis stipinkaulis bei suaugę galvinis ir kablinis kaulai. Paiešką atlikome duomenų bazėse internete („Pubmed“, „Scopus“, „Google Scholar“) ir aptikome 14 Irano mokslininkų straipsnių, kuriuose pranešta apie 21 pacientą (laikas nebuvo ribojamas). Apžvelgėme prieinamus klinikinius ir genetinius pacientų duomenis ir geografinę kilmę.

***Rezultatai.*** 14 straipsnių, kuriuose aprašyti 22 Irano pacientai, kuriems nustatytas EVC sindromas (taip pat ir mūsų pacientė), išleisti persų ir anglų kalbomis (7 persų kalba ir 7 anglų kalba). Visiems pacientams pasireiškė EVC būdingi simptomai, tačiau diagnozė nustatyta gana vėlai, 18 pacientų gimė santuokose, sudarytose tarp artimų giminaičių.

**Išvados. **Remiantis šiais tyrimais, galima nustatyti šeimas, kuriose gali pasireikšti ši genetinė mutacija. Tokias šeimas genetinėse konsultacijose iki santuokos tarp artimų giminaičių sudarymo galime įspėti apie galimus tokios santuokos pavojus. Be to, nustatę šeimas, kuriose gali kilti šių problemų, tokias kūdikių anomalijas galime nustatyti vykdydami atidesnį stebėjimą iki gimdymo.

**Raktažodžiai:** Ellis-van Creveld, Iranas

## Introduction

Chondroectodermal dysplasia (Ellis–Van Creveld syndrome (EVC)) (MIM#225500, ORPHA:289) is a rare type of skeletal dysplasia that is more common in but not limited to the Amish population. Less than 300 cases of the disease have been reported in English literature. The syndrome was first described by Ellis and van Creveld in 1940 [1]. In 1964, McKusick et al. reported 52 cases of EVC in the Old Amish population, 30 of whom died during the first year of life [2, 3]. It was the largest group of described patients with EVC. Studies have shown that the inheritance of this disease is autosomal recessive [4,5]. 

The exact prevalence of EVC is unknown and is estimated at approximately seven per million [6]. Some studies have reported the higher prevalence of the disease in Amish, Brazilians, Ashkenazi Jews, and Arab communities with high rates of consanguineous marriages [7,8,9]. EVC patients may have skeletal and extra skeletal problems due to chondral and ectodermal dysplasia, including disproportionate short‐limb dwarfism, narrow chest, cubitus valgus, genu valgus, postaxial polydactyly, sparse hair, multiple oral frenula, nail and teeth dysplasia, and congenital heart defects. Cognitive and motor problems have not been reported. About half of patients die by childhood, primarily due to heart disorders [10,11,12]. EVC is genetically heterogeneous; mutations in EVC (MIM#604831) and EVC2 (MIM#607261) genes have been reported in patients with this syndrome [13].

We describe a case of an adult female patient with EVC and review reported Iranian patients with this syndrome.

## Case presentation

The patient was a 30 years old woman who underwent surgical treatment at our center due to post-axial polydactyly. The patient stated a history of bilateral knee surgery due to bilateral valgus of the knee (proximal tibial and distal femoral osteotomy) ten years ago. The patient was born to a consanguineous marriage; her parents were first cousins. The patient also had a cousin (uncle’s from mother’s side daughter) with the same disease and the patient’s father mentioned that his aunts had similar symptoms. The patient’s parents were from Naghadeh county, West Azerbaijan province, in the northwestern part of Iran.

**Figure 1:**
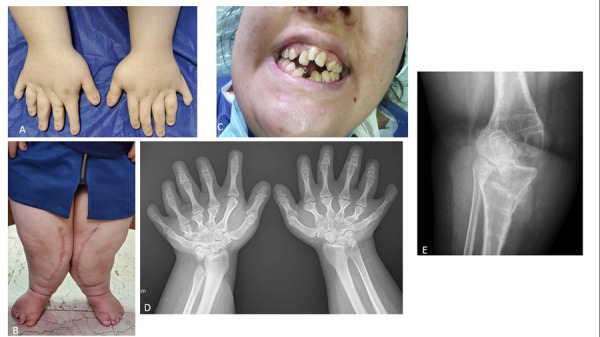
Clinical and radiologic manifestations of Ellis–van Creveld Syndrome. A: bilateral brachypolydactyly, hypotrophic fingernails. B: flat sole, pronated foot and hypotrophic nails. No polydactyly was seen in the toes. C: serrated incisal margins, dental transposition, diastema, conical teeth and enamel hypoplasia. D: radiographic features of upper extremity including postaxial polydactyly and polymetacarpia, enlarged distal radius and fusion of capitate and hamate. E: Knee valgus is evident despite previous surgery.

On examination, the patient was about 120 cm tall and had acromesomelic type dwarfism. She had bilateral polydactyly along with brachymetacarpia and hypotrophic fingernails (figure 1). Her finger and wrist movements were in the normal range. In the lower limbs, flat feet with pronated posture and hypotrophic nails without polydactyly were seen (figure 1). Oral examination revealed serrated incisal margins, dental transposition, a diastema, conical teeth and enamel hypoplasia (figure 1). The patient had undergone cardiovascular workup for previous surgeries and had no problems.

In the radiographic survey of upper extremity, postaxial polydactyly and polymetacarpia, enlarged distal radius and fusion of capitate and hamate were seen. Knee valgus was also evident despite previous surgery (figure 1).

## Discussion

Ellis–van Creveld syndrome (EVC) is a rare autosomal recessive disease. Patients with the EVC syndrome typically have a tetrad of features:

(1) Disproportionate dwarfism, (2) Bilateral postaxial polydactyly, (3) Hidrotic ectodermal dysplasia, and (4) Cardiac anomalies in 50-60% of the cases.

Histopathological evidence shows irregular chondrocytes in the metaphyses of long bones and, to some extent, metaphyses of the central vertebrae [14]. Chondrodysplasia includes bone growth disorders that cause symptoms such as short stature. Dwarfism in EVC patients resulting from a shortening of both ends of long bones may not be evident at birth. Ectodermal dysplasia affects tissues that originate in the ectoderm, such as hair, nails, and teeth. Congenital heart disease is present in about half of EVC patients and is the most important cause of morbidity in this disease. Some studies have reported the highest prevalence of the disease in Amish, Brazilians, Ashkenazi Jews, and Arab communities with high rates of consanguineous marriages [7,8,9].

14 Iranian papers in Persian and English have reported 21 patients with this syndrome so far (seven articles in Persian and seven articles in English) [15-28]. The details of the reported patients are described in Table 1. 

**Table 1.** Demographic, clinical and genetic data of 22 Iranian patients with EVC syndrome.

Author (Date of publication) [ref] (published language)	Number of patients reported	Phenotype	Patients’ place of residence	Patients’ age at the time of reporting	Genetic testing results	Family history
Afshar et al. (1998) [15] (Persian)	1	Disproportionate dwarfism, postaxial polydactyly and bra-chymetacarpia, dystrophic nails, cardiac anomaly, facial dysmorphism, oligodontia, multiple wide frenula, conical teeth	Not mentioned	10		
Bayani (1999) [16] (Persian)	1	Disproportionate dwarfism, postaxial polydactyly and bra-chymetacarpia, dystrophic nails, cardiac anomaly, narrow thorax	Guilan province (north of Iran)	Neonate (died)		5 previous abortions in mother
Baghdadi et al. (2001) [17] (Persian)	2	Disproportionate dwarfism, postaxial polydactyly and bra-chymetacarpia, multiple wide frenula, dystrophic nails, cap-itohamate fusion, genu valgum	East Azerbaijan province	15 and 11 (two brothers)		A died girl in 18 months of age with similar phenotype
Alizadeh et al. (2004) [18] (Persian)	1	Disproportionate dwarfism, postaxial polydactyly and brachymetacarpia, dystrophic nails, carpal fusion, multiple frenula, cardiac anomaly	Guilan province (north of Iran)	10		Non-Consan-guineous marriage
Mehralizadeh (2004) [19] (Persian)	1	Disproportionate dwarfism, postaxial polydactyly and brachymetacarpia, dystrophic nails, hydronephrosis, cardiac anomaly	Semnan province (central region of Iran)	neonate		Two previous infantile deaths and one abortion
Naseri et al. (2004) [20] (Persian)	3	Acromesomelic dwarfism, postaxial polydactyly and brachymetacarpia, hypoplastic nails, multiple frenula, narrow thorax	Khorasan province (eastern region of Iran)	All neonate		Two died, one from non-consanguineous marriage, previous similar child in one patient
Moham-madzadeh et al. (2005) [21] (Persian)	5	Acromesomelic dwarfism, postaxial polydactyly and brachymetacarpia, hypoplastic nails, genu valgum, multiple frenula, oligodontia, cardiac anomaly	Khorasan province (eastern region of Iran)	6, 8, 9, 11 years old and one neonate		Two patients were brother and sister, one patient was from Sistan Bal-uchestan province (south east region of Iran)
**Saneifard** et al. (2008) [22] (English)	1	Acromesomelic dwarfism, post-axial polydactyly and brachym-etacarpia, hypoplastic nails, genu valgum, multiple frenula, oligodontia, conical teeth, cardiac anomaly, narrow thorax	Fars Province (south region of Iran)	9		Nonconsanguine-ous marriage
Aminabadi et al. (2010) [23] (English)	1	Acromesomelic dwarfism, postaxial polydactyly and brachymetacarpia, hypoplastic nails, genu valgum, multiple frenula, oligodontia, conical teeth, serrated gingiva	East Azerbaijan province	5		Two previous abortions
Tahririan et al. (2014) [24] (English)	1	Acromesomelic dwarfism, postaxial polydactyly and brachymetacarpia, hypoplastic nails, oligodontia, cardiac anomaly	Isfahan province (central region of Iran)	2.5		
Alaee et al. (2014) [25] (English)	1	Acromesomelic dwarfism, postaxial polydactyly and brachymetacarpia, hypoplastic nails, genu valgum, oligodontia, cardiac anomaly	Golestan province (north eastern region of Iran)	3		
Nazemisal-man et al. (2016) [26] (English)	1	Acromesomelic dwarfism, postaxial polydactyly and brachymetacarpia, hypoplastic nails, genu valgum, cardiac anomaly	Zanjan province (north eastern region of Iran)	7		One abortion and one infantile death with similar phenotype
Eftekhari-yazdi et al. (2020) [27] (English)	1	Dwarfism, narrow thorax	Khorasan province (eastern region of Iran)	Fetus (terminated pregnancy)	One homozygote variant in EVC2 gene was identified in the fetus (NM_147127, c.942G>A, p.W314X).	One previous terminated pregnancy with similar phenotype,
Ghassemi et al. (2020) [28] (English)	1	Acromesomelic dwarfism, postaxial polydactyly and brachymetacarpia, hypoplastic nails, genu valgum, cardiac anomaly pectus excavatum, Phrygian cap gallbladder, liver hemangioma, polycystic ovarian disease	Khorasan province (eastern region of Iran)	40		Nonconsangui-neous marriage, one brother with similar phenotype.

The three most exciting issues are:

• The referral of most of these patients at older ages.
• The high rate of consanguineous marriages in the affected families.
• The geographical distribution of the patients’ place of birth or race and ethnicity.

Hospitals in Iran are organized into four levels based on the number of available beds and specialties. The first level includes local hospitals; the second level - hospitals under 100 beds; the third level - hospitals in provincial capitals; and the fourth level includes hospitals of the scientific hubs located in metropolises. Many reported patients have been from the third and fourth level hospitals of northern regions (including northeast and northwest) of Iran. These regions could be endemic regions for this genetic disease. However, this claim needs more studies by finding known families and genetic studies on them. 

Prenatal screening for fetal anomalies is a part of the prenatal care services that are recommended for all expecting mothers in Iran. Almost all (98%) pregnant women in Iran undergo ultrasound screening for fetal diseases at about 18 weeks of gestation. The primary purposes of this assessment are to estimate gestational age, diagnose multiparity and probable anomalies and control the fetus position. Abnormal ultrasound findings at 18 weeks of gestation in the fetus with Ellis–van Creveld syndrome include shortening of the long bones (femur, humerus, and tibia) below the fifth percentile for fetal age, narrow chest, postaxial polydactyly, and atrial septal defects. In patients with a familial history of the disease, genetic counseling before pregnancy is indicated. According to the current laws in Iran, termination of pregnancy before the ensoulment (4 months and 10 days of the fetus) is allowed when the mother’s life is in danger or the fetus has severe anomalies. Besides, the mother must have an approval of the judicial authorities. The judge usually has to obtain the official permission of three doctors to make such a ruling [13, 27]. Patients with the EVC syndrome typically have a tetrad of sonographic features:

(1) Disproportionate dwarfism, (2) Bilateral postaxial polydactyly, (3) Hidrotic ectodermal dysplasia, and (4) Cardiac anomalies in 50-60% of the cases.

Except for one case when after prenatal diagnosis pregnancy was terminated, all other patients did not have a history of prenatal care and diagnosis. Many patients had a family history of similar cases and were born to consanguineous marriages, but no genetic counseling was performed before the pregnancy. These problems were also present in our patient. Most of these patients came from rural areas. There is a national guideline for prenatal care and obstetrical services in Iran. According to this regulation, pregnant mothers in rural areas are monitored by rural health houses and are referred to higher levels if needed. Referrals for ultrasound screening by rural health centers are recommended but not mandatory. This can be the reason for not diagnosing some of these anomalies. Rural health centers are required to refer women who are at high risk for pregnancy and fetal complications to a higher level central health center (where a doctor is present). However, in many villages, pregnant mothers do not have the necessary cooperation. For this reason, more accurate follow-up of known cases of genetic diseases and consultation with them is recommended.

This study have investigated demographic, clinical and genetic data of described Iranian patients with EVC, identified some exceptional aspects as older age at diagnosis, high rate of consanguineous marriages, low rate of genetic testing and origins of most of the patients in the Northern region of Iran. Identifying families who are susceptible to the disease in their children and premarital genetic counseling as well as careful follow-up of pregnancies resulting from these marriages can help reduce the incidence of the disease. Having a national database of patients with genetic diseases in the country can help achieve this goal. Undoubtedly, to better comprehend the status of this disease, prospective studies are needed in cooperation with all medical universities in the country. Also, designing studies to investigate the common genetic mutation causing this disease in Iran will be helpful.
